# Semantic Interconnection Scheme for Industrial Wireless Sensor Networks and Industrial Internet with OPC UA Pub/Sub

**DOI:** 10.3390/s22207762

**Published:** 2022-10-13

**Authors:** Chenggen Pu, Xiwu Ding, Ping Wang, Shunji Xie, Junhua Chen

**Affiliations:** 1 School of Computer Science and Technology, Chongqing University of Posts and Telecommunications, Chongqing 400065, China; 2 Key Laboratory of Industrial Internet of Things & Networked Control, Ministry of Education, Chongqing University of Posts and Telecommunications, Chongqing 400065, China

**Keywords:** Industry 4.0, industrial wireless sensor networks, OPC UA, WIA-PA, publish/subscribe

## Abstract

In the Industry 4.0 era, with the continuous integration of industrial field systems and upper-layer facilities, interconnection between industrial wireless sensor networks (IWSNs) and industrial Internet networks is becoming increasingly pivotal. However, when deployed in real industrial scenarios, IWSNs are often connected to legacy control systems, through some wired industrial network protocols via gateways. Complex protocol translation is required in these gateways, and semantic interoperability is lacking between IWSNs and the industrial Internet. To fill this gap, our study focuses on realizing the interconnection and interoperability between an IWSN and the industrial Internet. The Open Platform Communications Unified Architecture (OPC UA) and joint publish/subscribe (pub/sub) communication between the two networks are used to achieve efficient transmission. Taking the Wireless Networks for Industrial Automation Process Automation (WIA-PA), a typical technology in IWSNs, as an example, we develop a communication architecture that adopts OPC UA as a communication bridge to integrate the WIA-PA network into the industrial Internet. A WIA-PA virtualization method for OPC UA pub/sub data sources is designed to solve the data mapping problem between WIA-PA and OPC UA. Then, the WIA-PA/OPC UA joint pub/sub transmission mechanism and the corresponding configuration mechanism are designed. Finally, a laboratory-level verification system is implemented to validate the proposed architecture, and the experimental results demonstrate its promising feasibility and capability.

## 1. Introduction

With the rapidly growing demand for wireless data transmission services in the industrial Internet of things (IIoT) [1,2], industrial wireless sensor networks (IWSNs) have been playing a significant role in the development of large-scale industrial networks and have become an efficient, cost-effective network solution for process control and industrial automation in factories [3]. The International Electrotechnical Commission (IEC) has released three international IWSN standards: WirelessHART [4], Wireless Networks for Industrial Automation Process Automation (WIA-PA) [5], and ISA100.11a [6]. Through these industrial wireless technologies, an IWSN can transmit commands to actuators or aggregate the data produced by wireless field devices in a factory. In the context of Industry 4.0, the massive data collected by field devices in an IWSN can effectively drive the development of industrial intelligent manufacturing and factory digital twin systems and play an important role in industrial decision-making [7]. The interconnection and interoperability between IWSNs and the industrial Internet can not only facilitate a seamless connection between the physical and information worlds and promote the development of IIoT in the industrial Internet [8] but also enable IIoT data sharing to enhance the potential value of field-level data [9].

All three IWSN technologies are based on IEEE 802.15.4 and form wireless personal area networks. The gateway of an IWSN is responsible for data collection from wireless devices and for the downward forwarding of commands to wireless devices. In real industrial scenarios, IWSNs are often connected to legacy control systems (e.g., supervisory control and data acquisition (SCADA) and human–machine interface (HMI)), through industrial wired network protocols (e.g., Modbus, Foundation Fieldbus, and Powerlink) via gateways [3]. However, complex protocol translation in the application layers is required in these gateways, and the three IWSN standards use different translation methods and implementation. This makes it difficult to integrate IWSNs into the industrial Internet quickly and efficiently. Meanwhile, although data can be transmitted between IWSNs and the industrial Internet through legacy wired industrial network protocols, semantic interoperability between IWSNs and the industrial Internet has not been fully investigated. To fill this gap, our study focuses on (1) realizing interconnection and interoperability between IWSNs and the industrial Internet through the Open Platform Communications Unified Architecture (OPC UA) and (2) creating a joint publish/subscribe (pub/sub) communication pattern between the two networks to achieve efficient transmission. Taking WIA-PA, a typical industrial wireless technology in IWSNs, as an example, we propose a method of efficiently connecting WIA-PA to the industrial Internet to maximize the potential of IIoT data.

As an open, platform-independent and service-oriented architecture specification, OPC UA supports the secure and reliable exchange of data, in the industrial automation space and other industries, and ensures the seamless flow of information among devices from multiple vendors [10]. OPC UA provides a flexible information security model that ensures a secure, reliable interaction of data and information between devices [11]. It is a widely accepted industrial standard that is supported by many credible universities and industrial companies [12]; it can provide a viable solution that will guarantee interconnection and interoperability between WIA-PA networks (or IWSNs) and the industrial Internet. There are currently two communication modes in OPC UA: client/server (C/S) and pub/sub. Compared with the C/S mode, which wastes resources and has low communication efficiency in resource-constrained networks, the pub/sub mode has a flexible communication structure, easy configuration, and real-time capability and support for many-to-many communication [13]. Pub/sub is also a communication mode of WIA-PA. As IWSN devices typically have limitations related to energy resources, processing capacity, and storage capacity, among others, the OPC UA pub/sub mode is highly suitable for WIA-PA networks. The combination of the WIA-PA and OPC UA pub/sub modes can utilize the information description ability of OPC UA and provide a safe, effective means of communication, enabling WIA-PA networks to connect to the industrial Internet. In addition, compared to the polling communication mode and node periodic reporting mode, in the publish/subscribe communication mode, only the events and data that are of interest need to be transmitted [14], which reduces unnecessary message transmission in the network, and, furthermore, reduces energy consumption and prolongs the network lifetime.

Most studies on WIA-PA and OPC UA focus on verifying and implementing their respective protocols. Numerous approaches have been proposed to enable connection between WIA-PA networks and the Internet. For instance, the authors in [15] proposed an IPv6 Internet access architecture and approach for WIA-PA networks. The method redefines the north–south interface for the protocol conversion module of a WIA-PA gateway supporting IPv6, and realizes a connection between the WIA-PA network and the IPv6 Internet. In addition, the method provides a valuable reference for the current paper, which aims to realize the interconnection between a WIA-PA network and the industrial Internet through OPC UA pub/sub. The authors in [16] analyzed the protocols of WIA-PA and Ethernet/IP and proposed a gateway that can transfer protocols between WIA-PA and Ethernet/IP and connect industrial wireless networks to the IPv4 Internet. Many researchers have applied Message Queuing Telemetry Transport (MQTT) to OPC UA pub/sub because it is simple, stable, open, lightweight, and easy to implement. The authors in [17] compared the performance of MQTT and OPC UA and concluded that MQTT is more suitable for distributing messages when a large number of subscribers receive the same topic. Therefore, MQTT can be utilized as an extension to OPC UA to support this use case, which we perform in the present paper. In [18], the authors proposed an OPC UA pub/sub implementation that uses the open62541 software development kit (SDK). They focused on implementing it in the binary message format with brokerless transport over a time sensitive network (TSN)-enabled Ethernet to create real-time networks.

Although the IWSNs and OPC UA pub/sub modes have been extensively studied in their respective fields of research [19,20], the combination of IWSNs and OPC UA pub/sub is hindered on the technical level and lacks mature solutions. Considering the abovementioned issues, this work proposes a communication architecture for IWSNs that is based on WIA-PA and OPC UA. This architecture can satisfy most requirements of IWSNs and combine WIA-PA pub/sub with MQTT broker-based OPC UA pub/sub to realize WIA-PA/OPC UA joint pub/sub. Specifically, aiming at the problem of data mapping between the WIA-PA device and OPC UA pub/sub server, a WIA-PA virtualization method for the OPC UA pub/sub data source is designed. Finally, an experimental system is implemented to demonstrate the feasibility and capability of the proposed communication architecture. To the best of our knowledge, this is the first work to deploy OPC UA pub/sub in a WIA-PA network. This paper is, thus, a significant reference for other industrial wireless technologies to connect to the industrial Internet through OPC UA and can also inspire more researchers to pursue this field. In summary, the main contributions of this work are summarized as follows:

(1) Taking WIA-PA, a typical wireless technology in IWSNs, as an example, we propose a communication architecture of WIA-PA/OPC UA joint pub/sub. This architecture can combine WIA-PA pub/sub with MQTT broker-based OPC UA pub/sub to realize the integration of the WIA-PA network into the industrial Internet.
(2) A WIA-PA virtualization method for OPC UA pub/sub data sources is designed to solve the data mapping problem between WIA-PA devices and OPC UA publisher/subscriber.
(3) To ensure the efficient messages exchange between OPC UA publisher/subscriber and WIA-PA networks, we design a WIA-PA/OPC UA joint pub/sub transmission mechanism and the corresponding configuration mechanism.
(4) An experimental system is implemented to evaluate the feasibility and capability of the proposed communication scheme. The results show that the proposed scheme performs well in terms of consumed memory, communication success rate, and publishing delay. Furthermore, in this study, the proposed scheme has strong protocol consistency.

The rest of this paper is organized as follows. Section 2 briefly introduces WIA-PA and OPC UA and analyzes the feasibility of their combination in different application scenarios. In Section 3, the proposed WIA-PA/OPC UA joint pub/sub communication architecture is described. The implementation and execution of the experimental system are described in Section 4. Section 5 summarizes the proposed method.

## 2. Overview of WIA-PA and OPC UA

### 2.1. WIA-PA

**Figure 1:**
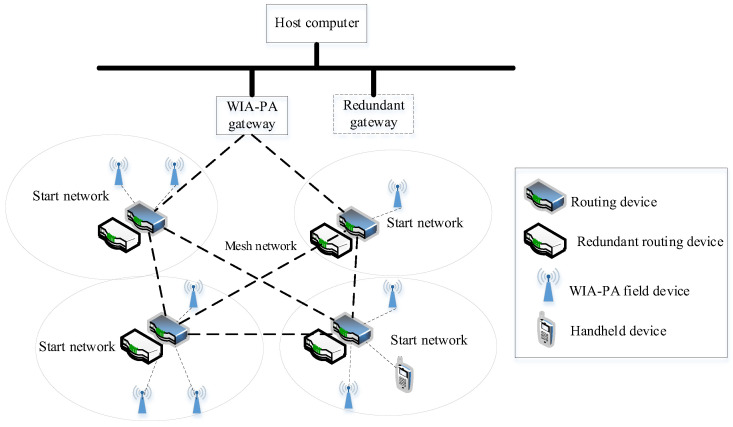
WIA-PA network topology.

WIA-PA is a wireless network protocol for industrial process measurement, monitoring, and control. It is based on IEEE 802.15.4 and is standardized and approved as IEC 62601 by the IEC [21]. Its topology is shown in Figure 1.

As shown in Figure 1, the basic elements in a WIA-PA network are (1) a host computer, (2) gateway devices, (3) routing devices, (4) field devices, and (5) a handheld device. To facilitate network management, WIA-PA also defines five logical roles, namely gateway, network manager, security manager, cluster head, and cluster member. The gateway is responsible for protocol data conversion between the WIA-PA network and other networks in the factory. The network manager manages and monitors the entire network. The security manager is responsible for key management and security authentication. The cluster head manages and monitors the field and handheld devices and aggregates and forwards data from cluster members or other cluster heads. Each cluster member acquires field sensor data and sends them to the cluster head.

The WIA-PA application layer includes the user application process (UAP) and the application sublayer (ASL). A UAP consists of one or more user application objects (UAOs). The properties of a UAO include object name, property name, property identifier, property data type, and supported methods. A UAO supports five operation methods for WIA-PA internal data: read, write, publish, report, and report ACK. To specify the access mode between UAOs, WIA-PA uses the virtual communication relationship (VCR), which distinguishes the paths and communication resources used by different UAOs. VCR_ID uniquely identifies each VCR. According to the supported applications, the VCR is divided into three modes: P/S mode (supports preconfiguration and periodic message transmission), R/S mode (supports aperiodic events and trend reporting), and C/S mode (supports WIA-PA aperiodic and dynamic pairwise unicast information transmission for WIA-PA devices).

### 2.2. OPC UA

OPC UA is a platform-independent, service-oriented interoperability technology that is standardized by the IEC and consists of 26 specifications and many companion specifications. It has two communication modes: C/S and pub/sub. In C/S, the client and server are deeply coupled. They must establish a direct connection and send requests and responses to each other. Compared with C/S, pub/sub has a flexible communication structure, easy configuration, and support for many-to-many communication.

**Figure 2:**
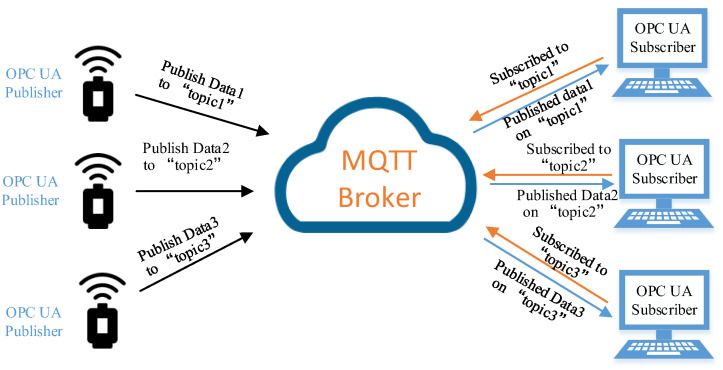
Broker-based OPC UA pub/sub communication architecture.

OPC UA pub/sub has three logical roles: publisher, subscriber, and message middleware. The publisher and subscriber are decoupled through the message middleware. They do not require direct information exchange but need to correctly parse and encapsulate message packets as required. Meanwhile, the OPC UA pub/sub specification defines brokerless and broker-based middlewares. With a brokerless middleware, an OPC UA publisher relies on the network infrastructure to deliver messages to one or more receivers. A broker-based middleware needs to use third-party messaging protocols, such as advanced message queuing protocol (AMQP) and MQTT. Compared with AMQP, MQTT is easier to implement and widely used in industrial automation application scenarios. Therefore, this paper uses MQTT as the WIA-PA/OPC UA joint pub/sub broker-based middleware. Figure 2 shows the OPC UA pub/sub communication architecture, which is based on the MQTT broker and has two message mapping methods: JavaScript Object Notation (JSON) and Unified Architecture Datagram Protocol (UADP).

### 2.3. Feasibility Analysis of Applying OPC UA Pub/Sub to WIA-PA

**Figure 3:**
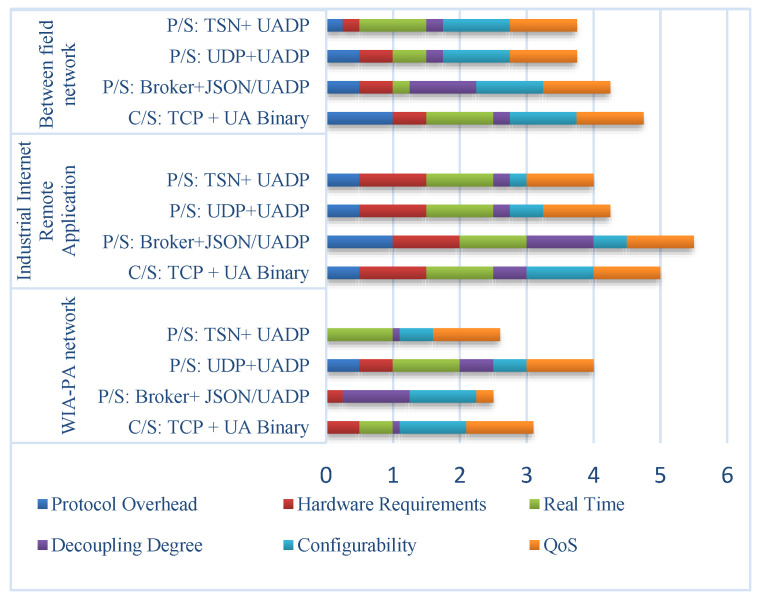
Feasibility analysis of OPC UA P/S applied to WIA-PA.

Eckhardt et al. [22] evaluated the applicability of OPC UA pub/sub to industrial automation from the perspective of industrial control. They provided empirical evaluation metrics, which served as a significant reference for the feasibility analysis of the combined WIA-PA and OPC UA in the current paper. Since WIA-PA is a mature industrial wireless technology, the architectures and protocols of WIA-PA and OPC UA should be considered in their combination. Based on the evaluation metrics and the evaluation criteria proposed in [22], aiming to combine WIA-PA and OPC UA, we qualitatively analyze the feasibility scheme of combining WIA-PA and OPC UA from the six indicators of protocol overhead, hardware requirements, configurability, quality of service (QoS), decoupling degree, and real-time performance. The quantitative scoring rules for the indicators are as follows: 1 means fully satisfied, 0.5 means generally satisfied, 0.25 means unsatisfied but can be optimized, and 0 means not satisfied at all. The analysis results are shown in Figure 3.

According to the analysis results in Figure 3, TSN+UADP performs better in certain indicators, such as real-time performance, configurability, and QoS. However, TSN requires dedicated hardware equipment and is, thus, difficult and expensive to implement. In the WIA-PA network communication scenario, since WIA-PA device resources are usually limited, it is difficult to transmit OPC UA messages, and implementation is difficult. In the cross-network communication scenario between field networks, OPC UA C/S provides services such as read, write, and subscribe and is, thus, more applicable than OPC UA pub/sub. However, compared with C/S, pub/sub has good applicability, especially in decoupling communication objects. In the communication scenario for industrial Internet remote applications, Broker+JSON/UADP has the best applicability except for configurability. It can fully satisfy the various indicators required by enterprise layer applications.

A broker in the OPC UA publish/subscribe model can decouple the communication between the entities in space, time, and synchronization. The publisher and subscriber do not have to be directly addressable, which makes the network architecture flexible, while there is no need for resource-constrained WIA-PA nodes to maintain address tables for subscribers. In addition, only the events and data that are of interest need to be transmitted to the broker, and the broker supports a one-to-many communication mode, which can reduce the number of messages to be transmitted and further reduce network conflicts and energy consumption. Hence, broker-based OPC UA pub/sub can meet most requirements of WIA-PA networks, industrial Internet remote applications, and cross-network communication between field networks.

In summary, OPC UA pub/sub is deployed in the WIA-PA scheme in this paper as follows. MQTT broker-based OPC UA pub/sub is the broker transport protocol, and JSON and UADP are the message encoding formats. Furthermore, the coupling of OPC UA and the WIA-PA network is realized through the WIA-PA gateway.

## 3. WIA-PA/OPC UA Joint Pub/Sub Scheme

### 3.1. System Architecture

**Figure 4:**
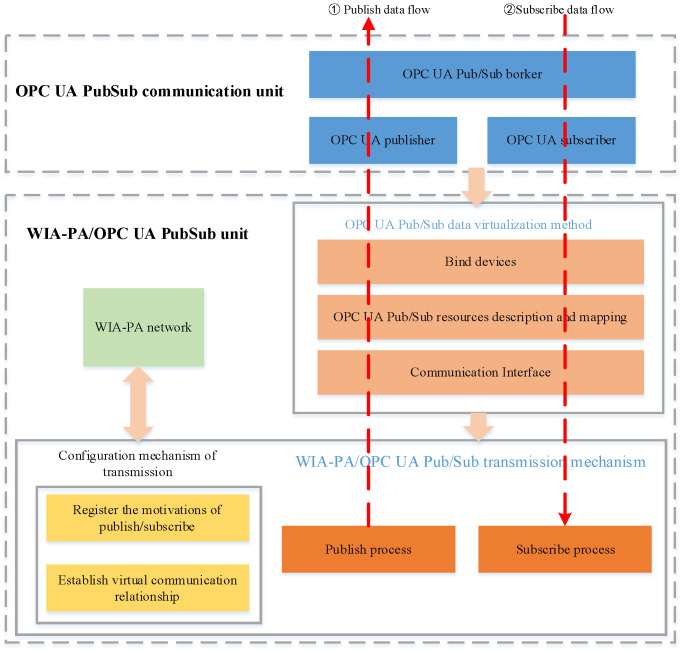
System architecture of WIA-PA/OPC UA joint pub/sub scheme.

In an IWSN, the computing resources and energy of sensor devices are relatively limited, whereas those of the gateway are generally greater. Therefore, the use of a WIA-PA gateway as the coupling point between WIA-PA and OPC UA can maximize its abundant computing resources and energy. The system architecture of the WIA-PA/OPC UA joint pub/sub is illustrated in Figure 4.

**Figure 5:**
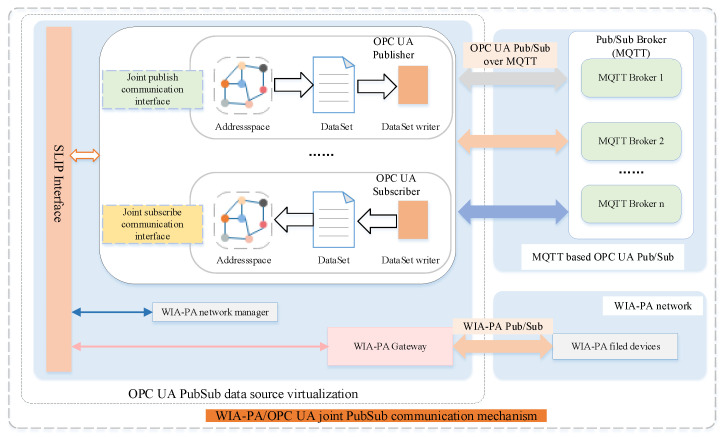
WIA-PA/OPC UA pub/sub software architecture.

The system architecture includes an OPC UA pub/sub communication unit and a WIA-PA/OPC UA joint pub/sub unit. The former realizes MQTT broker-based OPC UA pub/sub. The latter virtualizes the OPC UA pub/sub data source through a virtualization mechanism. In this way, the device binding and data resource mapping of the OPC UA publisher/subscriber are realized. Meanwhile, through the WIA-PA/OPC UA joint pub/sub transmission configuration mechanism, the WIA-PA network device can communicate with the OPC UA publisher/subscriber. Figure 5 depicts the software architecture of the WIA-PA/OPC UA joint pub/sub. The WIA-PA field devices, WIA-PA gateway, and MQTT-based OPC UA pub/sub unit jointly implement the WIA-PA/OPC UA joint pub/sub.

**Figure 6:**
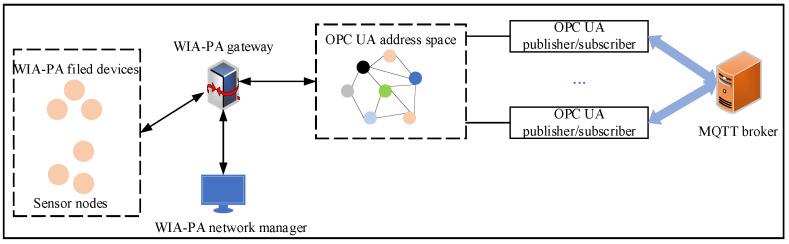
OPC UA pub/sub communication architecture for WIA-PA.

As shown in Figure 6, the system includes five entities: the field devices, WIA-PA gateway, WIA-PA network manager, OPC UA publisher/subscriber, and MQTT broker. These entities can be divided into two modules: the WIA-PA network module and the OPC UA pub/sub module. The former comprises the WIA-PA field devices, WIA-PA gateway, and WIA-PA network manager. The latter is composed of the OPC UA publisher/subscriber and the MQTT broker. The WIA-PA gateway is the communication bridge between the two modules. Since, in an IWSN, the computing resources and energy of sensor devices are relatively limited, whereas those of the gateway are generally greater, we use the WIA-PA gateway as the coupling point between WIA-PA and OPC UA to maximize its abundant computing resources and energy.

### 3.2. OPC UA Pub/Sub Data Source Virtualization Mechanism

WIA-PA and OPC UA have different pub/sub mechanisms and cannot be directly combined. Thus, a virtualization mechanism must be designed for the OPC UA pub/sub data source in the WIA-PA application layer. This mechanism is the basis of WIA-PA/OPC UA joint pub/sub communication. The method is to map the OPC UA pub/sub data source to the UAO of the WIA-PA application layer, such that the WIA-PA field devices obtain the data source of the OPC UA publisher/subscriber through the WIA-PA protocol. This method is divided into three steps: device interconnection, resource discovery, and OPC UA pub/sub data source mapping.

#### 3.2.1. Device Interconnection

**Figure 7:**
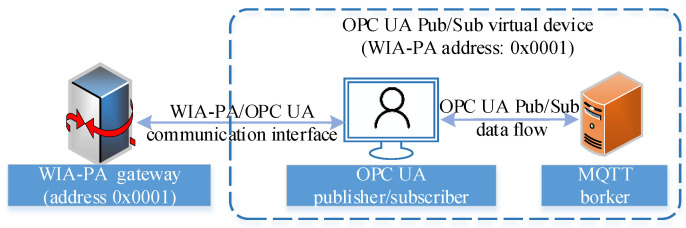
OPC UA pub/sub virtual device.

The device interconnection process is divided into two phases: WIA-PA gateway-based device interconnection and WIA-PA device address binding. In the former phase, the WIA-PA gateway performs resource discovery and communication transmission and realizes the connection between the OPC UA publisher/subscriber and the WIA-PA network. In the latter phase, the OPC UA publisher/subscriber and its corresponding broker middleware are virtualized as a virtual device in the WIA-PA application layer, which has a WIA-PA logical address and names the OPC UA pub/sub virtual device, as shown in Figure 7. The OPC UA pub/sub virtual device has a 16-bit WIA-PA short address. The WIA-PA field device can directly obtain the data source of the OPC UA publisher/subscriber through the short address.

#### 3.2.2. Resource Discovery

**Figure 8:**
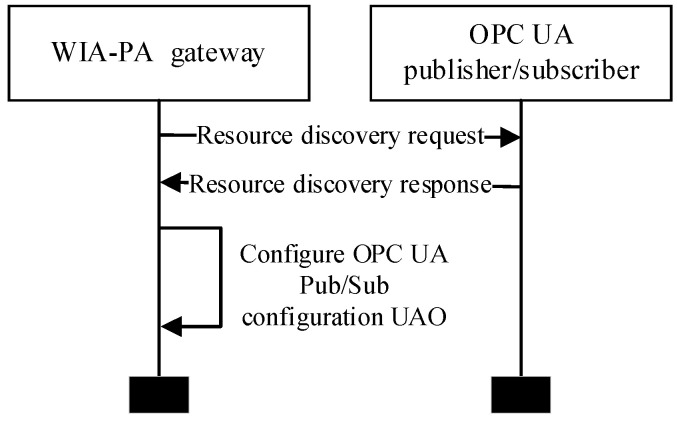
Resource discovery process.

The resource discovery process, which is shown in Figure 8, is initiated by the WIA- PA gateway by sending a request frame to the publisher/subscriber through the communication interface. After an OPC UA publisher/subscriber receives the request frame, it fills the response frame according to the frame header format and fills its pub/sub configuration information as the payload of the response frame. Then, the OPC UA publisher/subscriber sends the response frame to the WIA-PA gateway. After the WIA-PA gateway parses the response frame, it enters the OPC UA publisher/subscriber configuration information into an OPC UA pub/sub configuration UAO in its application. The OPC UA pub/sub configuration UAO is explained in detail in the Section 3.2.3.

#### 3.2.3. Resource Mapping of OPC UA Pub/Sub Data Source

After resource discovery, the discovered OPC UA pub/sub data source should undergo resource mapping. This process entails mapping the OPC UA publisher/subscriber information model to a pub/sub configuration UAO, which is in the WIA-PA gateway. Each OPC UA pub/sub configuration UAO corresponds to a publisher/subscriber. Therefore, through the WIA-PA protocol, the WIA-PA field devices can directly address the desired OPC UA data source.

The OPC UA publisher/subscriber information model is completely mapped to the UAO, and the payload becomes overly complex when WIA-PA devices communicate with the OPC UA servers. Therefore, in the design of the resource-mapping mechanism in this paper, the OPC UA pub/sub configuration information model is partially discarded to facilitate the data transmission of the WIA-PA field devices; only the necessary information models of these devices (e.g., publish dataset, pub/sub connection, and dataset writer) are retained.

Based on the simplified OPC UA pub/sub information model, we construct the communication parameters of the OPC UA pub/sub configuration UAO, which are divided into the public section, publisher section, and subscriber section.

- The public section contains the name, state, transport protocol, and connection address of the pub/sub connection, which are required by all publishers and subscribers.
- The publisher section includes the ID information of the publisher, writer group, and dataset writer; the data item and metadata of the published dataset; and the encoding format for message mapping.
- The subscriber section describes the publisher information subscribed by the OPC UA subscribers. To facilitate WIA-PA device communication, we add SubscriberID to identify the subscriber devices and DataSetReaderID to identify the dataset readers.

The parameters of the OPC UA pub/sub configuration UAO are classified as static and dynamic. The static configuration parameters are the various identification information of the OPC UA publishers and subscribers, such as PublisherID and SubscriberID, which are only used within the OPC UA applications and have little relationship with the WIA-PA field devices. We choose static parameters maintained by the WIA-PA gateway. The dynamic configuration parameters mainly are the connection protocol, connection address, topic, pub/sub time interval, and OPC UA message format, which are needed by the field devices to pub/sub data to the OPC UA server and are configured by the field devices before transmission. In this way, the protocol complexity of combining WIA-PA with OPC UA is effectively reduced, and the WIA-PA field devices can execute a pub/sub transmission without fully understanding the parameters of the entire OPC UA pub/sub.

### 3.3. WIA-PA/OPC UA Joint Pub/Sub Transmission Mechanism

**Figure 9:**
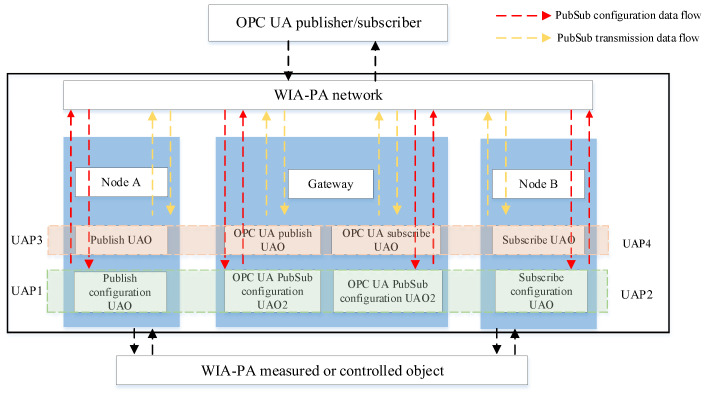
WIA-PA/OPC UA joint communication mechanism.

Figure 9 depicts the WIA-PA/OPC UA joint pub/sub transmission mechanism, including its configuration and transmission processes, which are implemented by the UAP in the WIA-PA network devices.

The functions of the different UAOs in Figure 9 are as follows:

- OPC UA pub/sub configuration UAO in the gateway. After discovering and mapping the OPC UA publisher/subscriber resources, the WIA-PA gateway establishes the pub/sub configuration mirror within it, namely the OPC UA pub/sub configuration UAO.
- OPC UA pub/sub message UAO in the gateway. It is responsible for parsing and forwarding the data sent by the WIA-PA field device. Each OPC UA pub/sub message UAO corresponds to an OPC UA pub/sub configuration UAO.
- Pub/sub configuration UAO in the field device. In the WIA-PA/OPC UA joint pub/sub configuration process, through the pub/sub configuration UAO, the field device registers the pub/sub operation parameters with the OPC UA pub/sub configuration UAO in the gateway and obtains the address of the OPC UA pub/sub message UAO in the gateway. The OPC UA pub/sub configuration UAO in the gateway and the pub/sub configuration UAO in the field device jointly realize the WIA-PA/OPC UA joint pub/sub configuration process.
- Pub/sub message UAO in the field device. It publishes the data collected by the WIA-PA field device to the OPC UA pub/sub message UAO in the gateway or receives the data from the OPC UA pub/sub message UAO in the gateway. The OPC UA pub/sub message UAO in the gateway and the pub/sub message UAO in the field device jointly realize the WIA-PA/OPC UA joint pub/sub execution process.

#### 3.3.1. WIA-PA/OPC UA Joint Pub/Sub Configuration Process

**Figure 10:**
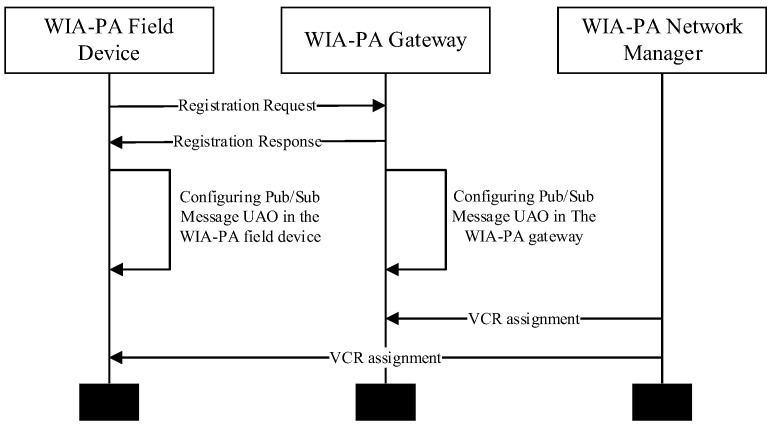
Time sequence flowchart.

The WIA-PA/OPC UA joint pub/sub configuration process can be divided into two parts: the pub/sub registration phase and the VCR establishment phase. Its time sequence flowchart is shown in Figure 10.

In the pub/sub registration phase, the pub/sub configuration UAO in the WIA-PA field device uses the “write” method to write the dynamic configuration parameters to the pub/sub configuration UAO in the gateway, including the broker address and protocol, topic, time interval, and message format (UADP or JSON), and finally enables the publication or subscription.

In the VCR establishment phase, the WIA-PA network manager allocates VCRs for the pub/sub message UAO in the WIA-PA field device and pub/sub message UAO in the gateway and configures the parameters, such as the UAO identifier and communication cycle.

#### 3.3.2. WIA-PA/OPC UA Joint Pub/Sub Transmission Process

**Figure 11:**
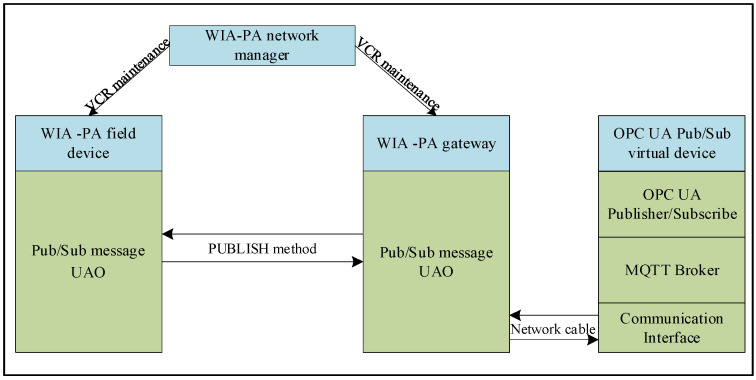
WIA-PA/OPC UA joint pub/sub interaction process.

The pub/sub transmission process between the WIA-PA field device and OPC UA publisher/subscriber entirely relies on the WIA-PA and OPC UA pub/sub modes, as shown in Figure 11. After the WIA-PA network manager establishes VCRs for the WIA-PA field device and OPC UA pub/sub virtual device, they can communicate.

In the publication of the data of the WIA-PA field device to the MQTT broker middleware, the WIA-PA gateway parses the received WIA-PA application layer protocol data and transmits them to the OPC UA publisher through the network cable. Next, the OPC UA publisher parses and enters the received data into the information space of the OPC UA publisher and then sends them to the MQTT broker middleware.

In the subscription process of the data from the MQTT broker middleware by the WIA-PA field device, after receiving the data subscribed from the MQTT broker middleware, the OPC UA subscriber enters the data into its information space and forwards them to the WIA-PA gateway through the network cable. The WIA-PA gateway encapsulates the received data into WIA-PA protocol data and sends them to the WIA-PA field device.

## 4. System Implementation and Execution

### 4.1. System Implementation

**Figure 12:**
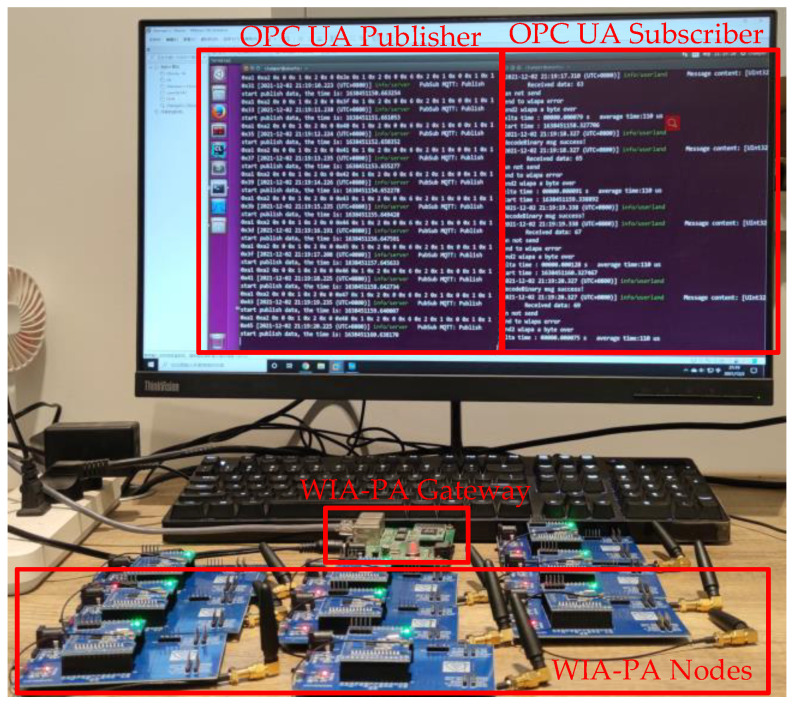
WIA-PA/OPC UA joint pub/sub experimental verification system.

On the basis of the system architecture (Figure 4) and software architecture (Figure 5), a laboratory-level WIA-PA/OPC UA joint pub/sub verification system is constructed, including WIA-PA field devices, a WIA-PA gateway, an OPC UA publisher, an OPC UA subscriber, and an MQTT broker, as shown in Figure 12.

In the verification system, embedded devices with STM32L152 (16KB SRAM and 128KB Flash) microcontroller run the WIA-PA wireless node protocol stack. An embedded device with RT5350 SoC (32MB SDRAM and 8MB NAND Flash) runs the WIA-PA gateway program. The WIA-PA nodes and the WIA-PA gateway form a star topology with the time slotted channel hopping function and the retransmission function (maximum number of retransmissions is three) enabled. The OPC UA publisher, subscriber, and MQTT broker run on a computer running Ubuntu 16.04 (Linux 4.4.0). The WIA-PA gateway connects to the computer through the network cable. The OPC UA publisher and subscriber are implemented using the open62541 SDK [23]. The Mosquitto [24] message broker software implements MQTT.

### 4.2. System Execution

This section describes system testing, which is divided into functional testing and performance testing. Functional testing includes WIA-PA/OPC UA joint publishing testing and subscription testing. Performance testing includes the memory occupied, the communication success rate, and the publishing delay.

#### 4.2.1. System Functional Testing

In WIA-PA/OPC UA joint publishing, the WIA-PA field device publishes data to the MQTT broker. In WIA-PA/OPC UA joint subscription, the WIA-PA field device subscribes to data from the MQTT broker. The WIA-PA/OPC UA joint publishing process and subscription process are detailed below.

- WIA-PA/OPC UA joint publishing process

The WIA-PA field device sends data to the OPC UA pub/sub virtual device in the WIA-PA gateway. Then, the OPC UA pub/sub virtual device parses the received data and forwards them to the OPC UA publisher through the network cable. The OPC UA publisher stores the received data, which are the data to be published, in its address space. The dataset writer adds properties to the data to be published and encapsulates them into the publishing dataset. The dataset writer group adds a publisher ID to the publishing dataset and encapsulates it into a message packet. Finally, the writer group encapsulates multiple message packets into a published message packet and then sends this packet to the MQTT broker. The dataset writer, dataset writer group, and writer group are logical concepts in the OPC UA publisher.

- WIA-PA/OPC UA joint subscription process

The OPC UA subscriber obtains the publisher-related communication parameters from the WIA-PA field device, such as Publisher ID, DataSetWriterID, and DataSetWriterGroupID, and then connects to the MQTT broker, according to the topic of the subscription message. After the OPC UA subscriber receives the subscribed data, it forwards them to the OPC UA pub/sub virtual device in the gateway through the network cable. Finally, the OPC UA pub/sub virtual device encapsulates the data into WIA-PA protocol data and sends them to the WIA-PA field device.

We built a laboratory-level experimental system to validate the WIA-PA/OPC UA joint pub/sub process. The experimental result shows that the proposed system can realize the WIA-PA/OPC UA joint pub/sub, supporting both the UADP and JSON message encoding formats and enabling the WIA-PA network to connect to the industrial Internet through OPC UA pub/sub.

#### 4.2.2. System Performance Testing

- Memory occupancy testing

**Figure 13:**
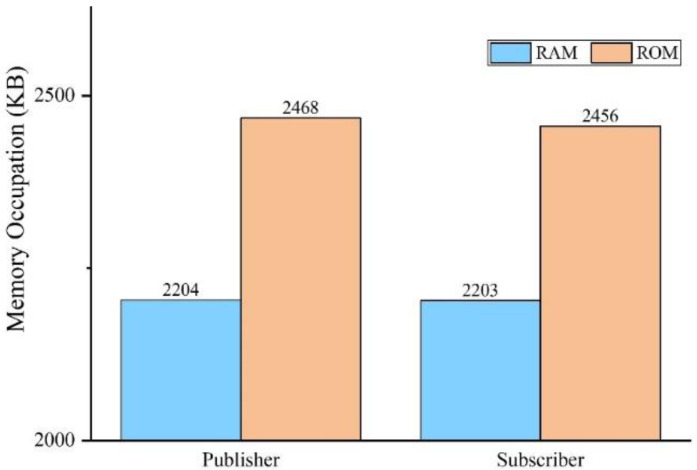
Memory occupancy of publisher and subscriber.

Memory occupancy testing involves the sizes of the read-only memory (ROM) and random-access memory (RAM) consumed by the OPC UA publisher/subscriber application. The test results are shown in Figure 13. The ROM and RAM consumption of both the publisher and subscriber is no more than 2500 KB and can, therefore, meet actual requirements.

- Communication success rate testing

**Figure 14:**
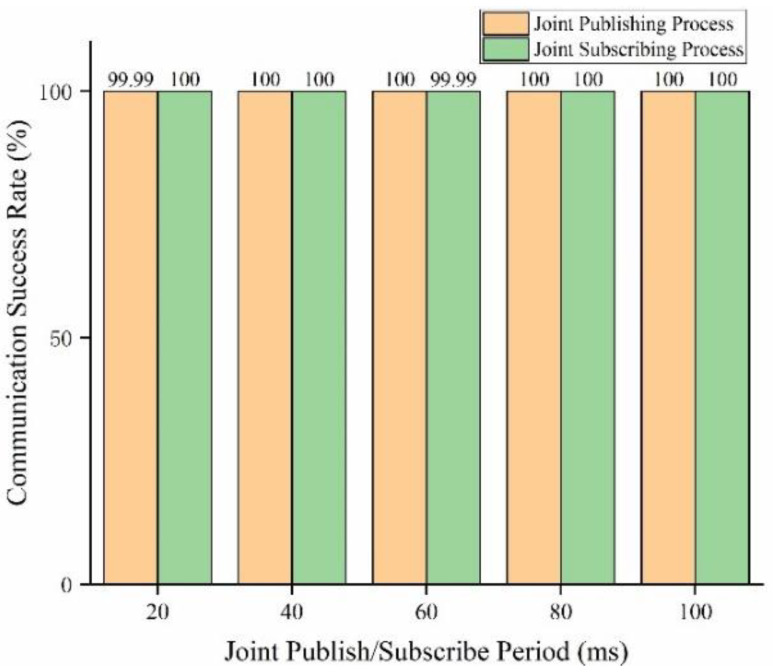
Communication success rate of WIA-PA/OPC UA joint pub/sub.

The communication success rate of WIA-PA/OPC UA joint pub/sub in different pub/sub periods (20–100 ms at intervals of 20 ms) is tested. The number of data packets sent by the WIA-PA field device or the MQTT broker is set to 10,000 in each period. In the joint publishing process, the success rate of publishing communication is the ratio of the number of messages received by the MQTT broker to the number of messages sent by the WIA-PA field device. Similarly, in the joint subscription process, the success rate of subscription communication is the ratio of the number of messages received by the WIA-PA field device to the number of messages sent by the MQTT broker. The test results are shown in Figure 14. The communication success rate of the joint pub/sub process is consistently above 99.99%, which fulfills the communication requirements of IWSNs. The test data contain very few failures. The reason for this is that the wireless link encounters interference or blockage, which leads to packet loss during the wireless transmission of WIA-PA.

- Publishing delay testing

**Figure 15:**
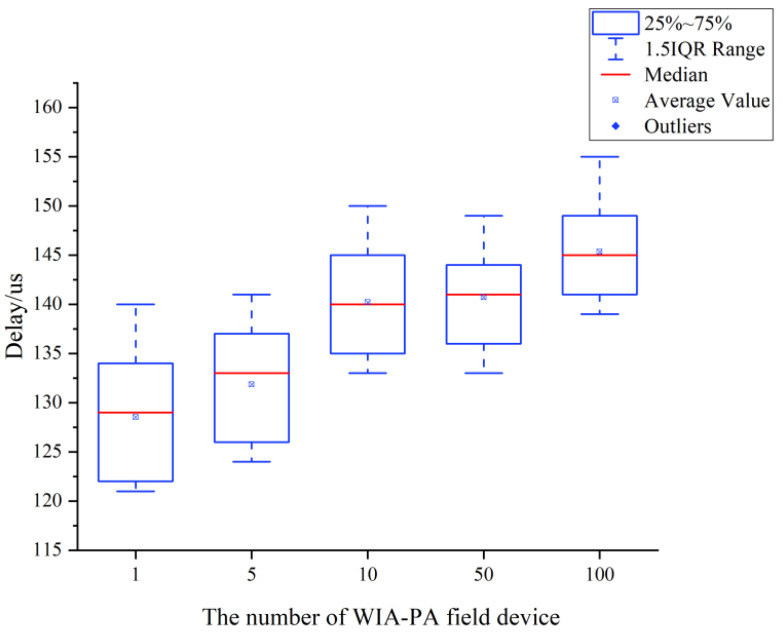
Joint publishing delay of WIA-PA field devices.

The joint publishing delay is defined as the time interval from when a message is received from the gateway’s wireless interface, after the gateway has processed the message and sent it to the OPC UA subscriber, to when the OPC UA subscriber receives this message. In cases with different numbers of field devices, we measure the publishing delay of the experimental system. The number of field devices is set to 1, 5, 10, 50, and 100. In each case, we count 100 sets of data and calculate the average. The results are shown in Figure 15. The proposed system maintains an average publishing delay of 125–150 μs under the different numbers of WIA-PA field devices. Hence, it can meet most communication requirements of IWSNs.

## 5. Conclusions

This paper addresses the problem of achieving interconnection and interoperability between IWSNs and the industrial Internet. Taking WIA-PA, a typical wireless technology in IWSNs, as an example, we propose an architecture of WIA-PA/OPC UA joint pub/sub. On the basis of the WIA-PA standard and OPC UA specification, we designed an OPC UA publisher/subscriber data source virtualization mechanism, the WIA-PA/OPC UA joint pub/sub transmission mechanism, and the corresponding configuration mechanism in the WIA-PA gateway. These mechanisms allow WIA-PA field devices and the OPC UA publisher/subscriber to communicate with each other. The WIA-PA gateway, which plays an important role in linking WIA-PA and OPC UA, was designed to ensure good coupling between WIA-PA and OPC UA and reliable data transmission. After simplifying the OPC UA pub/sub information model, we constructed UAOs for the OPC UA publisher/subscriber, which are used for parameter configuration and data transmission, according to the UAO design method provided by the WIA-PA standard. In addition, a laboratory-level WIA-PA/OPC UA joint pub/sub verification system was assembled to validate the proposed architecture. The experimental results of system execution showed that the proposed system architecture is feasible and performs well in terms of consumed memory, communication success rate, and publishing delay. It can meet most communication requirements of IWSNs.

In this study, the system architecture has strong protocol consistency; it does not modify the protocols of WIA-PA and OPC UA. This study serves as a valuable reference for connecting other industrial wireless technologies to the industrial Internet through OPC UA and can inspire more researchers to further advance this field. Future outlooks related to the proposed system architecture include the combination of the aggregation and disaggregation functions of WIA-PA to realize WIA-PA aggregation data transmission. We will add TSN technology to improve the real-time performance and reliability of the system, and we will also test the whole system in a real industrial scenario.
